# Society Proceedings

**Published:** 1902-09

**Authors:** 


### SOCIETY PROCEEDINGS.

### THE MISSOURI VETERINARY MEDICAL ASSOCIATION.

The eleventh annual meeting of this Association was called to order at 9 A.M., August 18, 1902, in the assembly hall of the Board of Education Building, northwest corner of Ninth and Locust Streets, St. Louis, Mo., by the Vice-President, Dr. Charles Doerrie, of Boonville, the President, Dr. J. W. Conaway, being absent on account of ill health.

The following members and visiting veterinarians were present: Drs. F. F. Brown, H. Bradley, C. W. Crowley, Charles Ellis, F. A. Goodbody, L. M. Klutts, B. F. Kaupp, R. A. Kammerer, D. F. Luckey, T. J. Menestrina, S. Stewart, A. Darling, H. B. Piatt, Ray. J. Stanclift, 8th U. S. Cavalry, J. M. Watson, W. H. Meadors, T. W. Scott, H. Timmermann, D. G. Painter, R. J. Sollberger, F. W. Hopkins, N. C. Powell, F. L. Combs, R. F. Eagle, J. J. Hougendobler, H. J. Hammerstein, D. C. Burnett, T. B. Pote, W. F. Heyde, and U. G. Houck.

The minutes of the previous meeting, held in Kansas City, October 22 and 23, 1901, were read and approved.

Under communications and correspondence, the Secretary read a letter from Dr. W. Horace Hoskins, of Philadelphia, Pa., in regard to attending the National Veterinary Association, to be held in Minneapolis, September 2, 3, and 4, 1902; also, a letter from Dr. E. Brainerd, of Memphis, Mo., sending his regrets of his inability to attend.

Vice-President Dr. Charles Doerrie appointed Drs. D. F. Luckey and L. M. Klutts on the Committee on Elections in the place of Drs. W. E. Martin and F. W. O'Brien, who were absent. Under the head of applications for membership the names of the following veterinarians were presented: Drs. A. Darling, St. Louis; Oscar Stuart, Florida; William McEachran, Louisiana; James Mahon, Carrollton; E. Brainerd, Memphis; F. A. Goodbody, St. Louis, and A. Trickett, Kansas City. Dr. D. F. Luckey moved that Dr. R. A. Kammerer cast the vote of the Association for the above applicants to become members. The motion was seconded and carried, and Dr. Kammerer cast the vote of the Association.

The election of officers was next in order. Dr. S. Stewart placed in nomination the name of Dr. D. F. Luckey, of Columbia, for President for the ensuing year. A motion was then made, which was seconded and carried, that Dr. Luckey be elected by acclamation. Dr. Charles Ellis then placed in nomination the name of Dr. R. A. Kammerer, of St. Louis, for Vice-President. Dr. H. B. Piatt made a motion that the Secretary be instructed to cast the vote of the Association for Dr. Kammerer for Vice-President for the coming year; motion seconded and carried. Dr. D. F. Luckey placed in nomination the name of Dr. B. F. Kaupp, of Kansas City, for re-election for Secretary-Treasurer for the coming year. A motion was made, seconded, and carried that the Secretary be re-elected by acclamation. The Secretary then read a report for the Secretary and Treasurer for the past year. Dr. S. Stewart made a motion that the reports be accepted as read, which was seconded and carried.

Dr. D. F. Luckey then gave a good and interesting address upon the subject of State veterinary legislation, pointing out some of the obstacles in the way of securing needed veterinary legislation in the State of Missouri. Moved by Dr. S. Stewart, that the President appoint a committee of three on State veterinary legislation, and to make an effort to secure needed legislation during the next session of the State Legislature. The motion was seconded by Dr. Charles Ellis and carried. It was then moved, seconded, and carried that the meeting adjourn for luncheon.

At 2 P.M. the meeting was called to order by the Vice-President, Dr. Doerrie, when the following programme was presented: "Puerperal Mania. in Bovines," by Dr. E. M. Nighbert; "Complications in Colic," by Dr. S. Stewart. Dr. Stewart went thoroughly into the subject, and the paper brought out a good discussion. Among those that entered into the discussion were Drs. A. Darling, L. M. Klutts, D. F. Luckey, Charles Ellis, and others. The next paper on the programme was "My Practical Experience Since Our Last Meeting," by Dr. L. M. Klutts, which paper was interesting and brought out a good discussion. Following these papers a number of interesting cases were reported. At 6 o'clock it was moved, seconded, and carried that the meeting adjourn to the northeast corner of Sixth and Chestnut Streets, where the veterinarians present were royally banqueted by the veterinarians of St. Louis.

At 8 o'clock P.M. the meeting was again called to order, when the following programme was presented: "Differential Diagnosis of Actinomycosis and Tuberculosis," by Dr. R. F. Eagle. After the reading of this valuable paper a motion was made by Dr. S. Stewart, which was seconded and carried, that the courtesies of the floor be extended to the visiting veterinarians. After a thorough discussion of this paper the following were presented: "Poisoning by Nightshade," by Dr. F. F. Brown; "Caseous Lymphadenitis in Sheep," by Dr. S. L. Shaw. Following these papers many interesting cases were reported, after which Dr. Charles Ellis, of St. Louis, moved that the following resolutions be adopted, which was seconded by Dr. A. Darling, and carried:

WHEREAS, The A. V. M. A. has established the precedent of holding meetings in cities where national and international fairs are held; and

WHEREAS, An international fair will be held in St. Louis in 1904; and

WHEREAS, No meeting of the A. V. M. A. has been held in the State of Missouri; and

WHEREAS, The foregoing circumstances make St. Louis the logical city for holding the Association's meeting in 1904; therefore, be it

*Resolved*, That the Missouri Veterinary Medical Association now in session invites the American Veterinary Medical Association to meet in St. Louis in 1904.

A motion was then made by Dr. D. F. Luckey, which was seconded by Dr. Charles Ellis and carried, that the Association extend a vote of thanks to Dr. R. F. Eagle for his valuable paper.

It was moved, seconded, and carried that the next meeting be held in Windsor, Mo., in 1903, at such time as the officers of the Association and Executive Committee may decide.

A motion was made by Dr. S. Stewart, which was seconded and carried, that a vote of thanks be extended to the local committee and other veterinarians of St. Louis for their hospitality.

At 11 o'clock, by a motion which was seconded and carried, the meeting adjourned to meet at Manchester Avenue and King's Highway, at 9 A.M., August 19th.

On Tuesday, August 19th, at 9 A.M., a clinic was held at Dr. R. A. Kammerer's hospital. The excellencies of the Kansas City Veterinary College operating-table were demonstrated by Drs. F. F. Brown and S. Stewart. Many operations were performed, which were participated in by Drs. D. F. Luckey, Ray. J. Stanclift, F. F. Brown, L. M. Klutts, and others. At 1 o'clock the clinic adjourned to luncheon, which was given by the St. Louis veterinarians, after which the afternoon was spent in examining interesting cases presented at the clinic.

The meeting was then adjourned to meet in Windsor, Mo., in 1903.

B. F. KAUPP,
Secretary.

### THIRTY-NINTH ANNUAL MEETING OF THE AMERICAN VETERINARY MEDICAL ASSOCIATION.

*Minneapolis, Minnesota, September 2 to 5, 1902.*

### SOME THOUGHTS—MINNEAPOLIS MEETING.

THE official records show an attendance of about one hundred and twenty-five members, which is somewhat in excess of the Atlantic City meeting; so, in point of numbers, the Minneapolis meeting was a success. The papers and discussions were fully up to the standard of past performances. It should be the ambition of all the members to have presented to the meetings papers of high merit, and to keep the discussions free from irrelevant and worthless matter. We should aim to make the printed proceedings of the highest possible merit, for the Association, and in turn the profession, will be judged largely by the character of the printed proceedings of the annual meetings.

The members were not prompt in getting into the convention hall so that the meetings might begin on schedule time. The entire time of the meeting provided for by the programme could easily be utilized in attending to the business and presenting the literary part of the programme. When we get behind time everyone gets nervous, and many do not enter into the discussion because of lack of time. The forenoon session of the first day was abandoned because a large number of members were delayed in their arrival owing to the lateness of the train. I am sure that these members, as well as others who did not suffer the misfortune of such an annoying delay, have fully resolved that on subsequent occasions they will leave home at a time sufficiently in advance of the meeting to permit of their arrival in due time, even if their train is several hours behind time. This is a praiseworthy resolve.

The sessions of the Executive Committee, held during the meeting, are usually extended considerably beyond the time set for opening the convention, and in this way the whole convention body is kept from beginning its work. These delays could be prevented if the Executive Committee would, whenever possible, hold its sessions in the evening, or, if it must have a morning session, by beginning on time and adjourning at the time scheduled for opening the session of the convention. This is a reform that is much needed.

The abolition of smoking in the convention hall during the sessions of our Minneapolis meeting was a very commendable act, and one which is doubtless approved by the smokers themselves.

The convention halls used for some of our recent meetings have not been as well adapted for the purpose as is desirable. The success of the meeting depends largely upon the kind of hall we have. The members will not remain in a disagreeable place, and visitors will not venture into it. It would be better, if necessary, to pay a moderate sum for the proper kind of room than to accept an undesirable one free of cost. The committee-in-charge should, at future meetings, make sure to obtain a suitable hall. The usual hotel parlor or ordinary is in most cases undesirable. The room should be remote from street or other noises, should be well ventilated and cool, lighted from *both* sides or overhead, and not from in front or behind, and be large enough. Better that it should be too large than too small. If possible, the place of entrance should be from the centre behind and not from one corner, for it is well known that, if the entrance is at one corner, people will crowd about the entrance and will not move over to the far side, even if there are vacant seats there.

Our meetings are becoming better every year, and if each member will begin as soon as one meeting is over to make plans whereby he can enhance the excellence of the next one, we will be assured of a rapid and healthy growth of our organization. With a membership of over five hundred active workers there is no reason why the American Veterinary Medical Association should not take high rank among the scientific bodies of America. It should be our aim to see that it does.

JOHN J. REPP,
Secretary A. V. M. A.

### ATTENDANCE LIST.

The following members and visitors were present during the various sessions of the American Veterinary Medical Association :

Members. Drs. Adams, Annand, Ackerman, Anderson, F. E.; Abele, Ainsworth, Baker, A. H.; Beckett, Behnke, Bell, R. R.; Brenton, Butler, G. W.; Butler, J. S.; Butler, Tait; Brimhall, Burnham, Cooley, Cotton, C. E.; Cotton, T. B.; Crawford, Dalrymple, Dougherty, Dunphy, Ellis, Charles; Fischer, Gay, Gibson, Glennon, Gohn, Gould, J. N.; Gould, J. W.; Harris, Heck, Hernsheim, Hinman, Hoskins, Hughes, Hunter, Jakeman, Jewell, Johnson, Jopling, Kelly, Wm. H.; Knight, Knowles, Leech, Lowe, Wm. H.; Loveberry, Lyford, Lyman, R. P.; McInnes, McKenzie, McNeall, Marshall, Martin, J. W.; Mayo, Merillat, Monsarrat, Moore, R. C.; Quitman, Norton, Pearson, Peters, A. T.; Piatt, Pierce, B. D.; Rayen, Ranck, Repp, Reynolds, Robertson, J. L.; Robinson, Ruhl, Ryan, Ryder, Salmon, Schaefer, Schwarzkopf, Smith, F. E.; Schmitt, Shepard, Sprague, Stanclift, Stewart, Stringer, Thomas, Torrance, Walrod, Ward, Winchester, Witte, White, G. R.; Whitbeck, Williams, Young, Youngberg—95.

Members Reinstated. C. A. Clinton, A. A. Keys—2.

Members-elect. Drs. A. Bostrum, M. V. Byers, J. W. Cook, S. A. Coxe, J. H. Crawford, John H. Gould, J. W. Haxby, W. S. Henderson, C. J. Hinckley, W. C. Holden, F. A. Ilstrup, H. Jensen, F. E. Lambrecht, G. Lames, R. La Pointe, H. C. Lyon, W. A. McClanahan, D. M. McDonald, G. McGillivary, J. H. McLeod, Peter Malcolm, Edward L. Moore, H. F. Palmer, Calvert H. Playdon, H. A. Presler, Richard Price, F. A. Rich, C. J. Rhodes, J. G. Rutherford, George A. Scott, J. A. Scott, L. U. Shipley, S. P. Smith, C. E. Stewart, Arthur Trickett—35.

Delegate. Pennsylvania State Veterinary Medical Association, Dr. J. C. Foelker, Allentown.

Visiting Veterinarians. Illinois: F. H. Ames, Canton; W. H. Welch, Lexington; F. C. Grayson, Paxton; J. T. Nattress, Delavan.

Iowa: B. Harmon, Decorah; W. R. Fullerton, Dubuque; H. J. Hagerty, Dubuque; H. L. Stewart, Lacona; F. M. Roys, Manning; J. J. Richardson, Marcus; H. M. Gillian, Mason City; M. F. Leffingwell, Northwood; N. A. Kippen, Riceville; C. E. Narey, Spirit Lake.

Kansas: E. F. McGraw, Fort Scott.

Massachusetts: William M. Simpson, Malden; William Simpson, Springfield.

Minnesota: T. Falconer, Alexandria; M. S. Whitcomb, Austin; J. J. McLaughlin, Blue Earth; Wm. Soneral, Cambridge; R. Kjerner, Chatfield; H. Langerin, Crookston; J. J. Findlay, Duluth; John McKay, Duluth; L. Hay, Faribault; C. S. Shore, Lake City; H. C. Peters, Litchfield; M. J. Sexton, Minneapolis; J. W. Golden, Redwood Falls; J. Butters, Renville; John P. Graff, New Ulm; E. T. Frank, Warren; C. W. Bigarel, Wendell; Oscar Rydell, Wheaton.

Michigan: D. S. De Wolf, Hart; W. W. Conkey, Grand Rapids.

New Jersey: W. Runge, Newark.

North Dakota: W. S. Stinson, Crystal.

Ohio: Gilbert Hess, Ashland; P. A. Dillahunt, Springfield.

Ontario: Thomas Thacher, Renfreu.

South Dakota: J. P. Foster, Selby.

Wisconsin: S. Beattie, Madison; H. L. Russell, Madison; B. L. Clark, Monticello; C. E. Evans, Racine; D. Roberts, Waukesha; E. D. Roberts, Janesville—48.

Lady Visitors. Illinois: Mrs. A. H. Baker, Chicago; Mrs. W. W. Dixon, Chicago; Mrs. A. Eger, Chicago; Mrs. Joseph Hughes, Chicago; Mrs. J. F. Ryan, Chicago; Mrs. H. C. Peters, Litchfield.

Indiana: Mrs. C. B. Ainsworth, Greensburg.

Iowa: Mrs. J. I. Gibson, Denison; Mrs. Wm. A. Heck, Maquoketa; Mrs. C. J. Hinckley, Odebolt; Mrs. G. A. Johnson, Sioux City.

Kentucky: Mrs. D. A. Piatt, Lexington; Miss Piatt, Lexington.

Manitoba: Mrs. F. Torrance, Winnepeg.

Massachusetts: Mrs. A. M. Haley, Reading.

Michigan: Mrs. S. Brenton, Detroit; Mrs. Horace M. Gohn, St. Johns.

Minnesota: Mrs. A. Youngberg, Detroit; Miss A. May, Laseur; Mrs. H. C. Peters, Litchfield; Mrs. J. G. Annand, Mrs. S. D. Brimhall, Mrs. C. E. Cotton, Mrs. C. C. Lyford, and Miss Lyford, of Minneapolis; Miss Nellie Carroll, St. Anthony Park; Mrs. M. H. Reynolds, St. Anthony Park; Miss Florence Burnham, Stillwater; Mrs. E. T. Frank, Warren; Mrs. J.

A. Scott, Waverly; Mrs. G. Ed. Leech, Winona; Mrs. J. N. Gould, Worthington.

Missouri: Mrs. C. H. Jewell, Kansas City; Mrs. S. Stewart, Kansas City.

Montana: Mrs. M. E. Knowles, Helena; Mrs. O. Schwartzkopf, Fort Assiniboin.

Nebraska: Mrs. A. T. Peters, Misses Antoinette and Florence Peters, and Mrs. W. A. Thomas, of Lincoln; Mrs. V. Schaefer, Tekamah.

New York: Mrs. E. B. Ackerman and Mrs. R. R. Bell, Brooklyn; Mrs. J. E. Ryder, New York City.

Ohio: Mrs. Flora A Cooley, Cleveland; Mrs. Paul Fischer, Columbus; Mrs. T. Bent Cotton, Mt. Vernon.

Pennsylvania: Mrs. Emma Brooks, Mrs. W. H. Hoskins, Mrs. C. J. Marshall, and Miss Pearson, of Philadelphia.

South Dakota: Mrs. J. P. Foster, Shelby.

Tennessee: Mrs. W. C. Rayen, Nashville.

Wisconsin: Mrs. G. W. Butler, Eau Claire; Mrs. D. Roberts, Waukesha.

Other Visitors. District of Columbia: E. V. Wilcox, Washington.

Illinois: J. C. Clinton, W. W. Dixon, Alex. Eger, V. E. Kover, D. E. Osgoaby, and J. L. Schmidt, of Chicago.

Manitoba: J. B. Leeson, M.D., Brandon.

Minnesota: J. O. Comes, V. M. Connell, M.D., and J. C. Smith, of Minneapolis; Robert S. Taylor, F. D. Ketchum, and B. W. Kirby, of St. Paul; Jared Burton, Wheaton.

Missouri: E. Lee, Chillicothe.

Nebraska: J. C. Boyd, Omaha.

Pennsylvania: H. P. Brooks, Philadelphia.

Wisconsin: Master Butler, Eau Claire; F. E. Perkins, Ellsworth.

Iowa: Master Joe Heck, Maquoketa.

New Members: M. C. Baker, 194 Milton St., Montreal, Quebec; J. W. Beckwith, Shullsburg, Wisconsin; A. Bostrum, Minden, Nebraska; Samuel Burrows, Veterinary Hospital, U. of P., Philadelphia, Pa.; Fred F. Bushnell, Winsted, Connecticut; M. V. Byers, Osceola, Nebraska; Robert E. Cochrane, 450 Greenbush St., Milwaukee, Wisconsin; J. W. Cook, Duluth, Minnesota; J. H. Crawford, Harvard, Illinois; S. A. Coxe, Brandon, Manitoba; J. M. Creamer, Portland, Oregon; J. W. Dunham, Fargo, North Dakota; Otto Faust, 211 Union St., Poughkeepsie, New York; J. H. Gain, Experiment Station, Lincoln, Nebraska; J. H. Gould, Fairmont, Minnesota; John C. Hargrave, Medicine Hat, N. W. Territory, Canada; E. T. Harrington, 873 Broadway, S. Boston, Mass.; J. W. Haxby, Villisca, Iowa; W. L. Henderson, Carberry, Manitoba; C. J. Hinckley, Odebolt, Iowa; W. C. Holden, Delphos, Ohio; F. A. Ilstrup, Willmar, Minnesota; H. Jensen, Weeping Water, Nebraska; Geo. A. Knapp, Millbrook, Dutchess Co., New York; Morgan B. Lamb, Pullman, Washington; T. Lambrechts, Montevideo, Minn.; G. Lames, Dysart, Iowa; R. Le Pointe, Laseur, Minn.; H. C. Lyon, Hutchinson, Minn.; W. A. McClanahan, Redding, Iowa; D. M. McDonald, Brainerd, Minn.; Geo. McGillivray, Spring Valley, Minn.; J. H. McLeod, Charles City, Iowa; E. Makins, Jr., Abilene, Kansas; Peter Malcolm, New Hampton, Iowa; A. W. Miller, S. Omaha, Nebraska, Box 58; B. O. Minge, 147 Court St., Memphis, Tenn.; E. L. Moore, Brookings, South Dakota; H. F. Palmer, 608 Champlain St., Detroit, Mich.; Joseph W. Parker, San Antonio, Texas; Adolph J. Pistor, Newark, N. J.; C. H.

Playdon, Reading, Mass.; H. A. Pressler, Fairbury, Ill.; Richard Price, St. Paul, Minn.; C. J. Rhodes, Beloit, Wisconsin; F. A. Rich, Burlington, Vt.; J. J. Riordan, Beverly Farms, Mass.; J. G. Rutherford, Ottawa, Ont.; Geo. A. Scott, Independence, Iowa; John A. Scott, Waverly, Minn.; J. N. Sheppard, Langdon, North Dakota; L. U. Shipley, Sheldon, Iowa; S. P. Smith, Cando, North Dakota; U. S. Springer, Grand Rapids, Mich.; Harry F. Steele, Fort Sill, Oklahoma; C. E. Stewart, Chariton, Iowa; W. Swenerton, Carberry, Manitoba; R. H. Treacy, Bismarck, North Dakota; Arthur Trickett, 2417 Grand Ave., Kansas City, Mo.; Harry W. Watson, Haverhill, Mass.; A. L. Wood, Prairie City, Iowa.

Reinstatements: C. A. Clinton, Laurens, Iowa; A. A. Keys, 82 S. 8th St., Minneapolis, Minn.

### Report of Dr. C. J. Marshall,

STATE SECRETARY FOR PENNSYLVANIA.

The veterinary profession of Pennsylvania is made up of about 1700 registered men; nearly 300 of this number are college graduates. About 1000 names on our registration list were placed there improperly, and have ceased to have any identity with our profession. We are fairly well organized, and are working in harmony on all legislative and professional subjects. There is one State organization composed of about 200 working members, which represents all sections of the Commonwealth. There are, also, several energetic local societies. The State Association reached its twenty-first birthday last year. It attained its majority with a rich inheritance of perseverance and integrity from its founders, who are still guarding its interests as only thoughtful and loving parents can do. In the A. V. M. A. we find enrolled twenty-seven members from Pennsylvania. Of the 300 men eligible we hope soon to see a much larger percentage belonging to the National Association.

The Keystone Veterinary Medical Association meets in Philadelphia the first Tuesday evening of each month. At present it is trying to devise a plan for the improvement of the milk-supply to our hospitals and public institutions. A circular letter was sent out last winter to the different stewards or managers, and it was found that very little attention was being paid to the inspection of milk, the system of handling it, or the cleanliness of the herds and dairies supplying it.

The State Board of Veterinary Medical Examiners has been established seven years. In this short time it has demonstrated its usefulness. The records show each year that the young men presenting themselves for the examinations are much more thoroughly prepared for their work, and that better educated and more scholarly young men are entering our profession. This board is trying at present to get an Act passed by the Legislature whereby a re-registration will be made necessary. This appears to be the only plan by which the registration lists can be freed from the names of many persons who have no connection with our profession. This board has secured convictions against three illegal practitioners, and has three more cases in the courts at present. Most of the complaints brought before the board are adjusted peaceably. The Secretary attends personally and promptly to all complaints, and has the faculty of bringing about a settlement usually without legal assistance. His term of office expired last year, and the Governor recognized his usefulness by reappointing him, which action met with the unqualified approval of the entire profession.

The State Live-stock Sanitary Board of Pennsylvania was organized in 1895. Its thorough organization was brought about largely through the efforts of our State Veterinarian, whose tact, scientific skill, and indomitable perseverance have made it the admiration of our profession. We wish that every State in the Union had a similar board equally as well organized and working as harmoniously for the good of our live-stock interests. The little opposition to the board for the first few years has gradually subsided, until at present very little criticism is heard from any source. At present the board is trying to devise plans whereby cattle reacting to the tuberculin test and showing no visible signs of disease can be utilized with less loss to the owner and State than is necessary under the present regulations. It has under observation a good-sized herd of reacting animals, and is studying the sanitary precautions necessary to handle such a herd, and also the best way to dispose of its products.

The laboratory of the State Live-stock Sanitary Board is one of the most complete of its kind. All of the tuberculin, mallein, and most of the vaccines used in the State are manufactured in this laboratory and distributed among our profession free of charge. This alone represents a sum of money which would cost the State quite as much as it does to run the laboratory, yet its principal value is for research and diagnostic purposes. In the lines of research work a method is being tested for immunizing cattle against tuberculosis by a process of vaccination with attenuated cultures. For diagnostic purposes the laboratory has become a necessity. Special shipping boxes are deposited with veterinarians in different sections of the State with directions for packing and shipping specimens. These specimens are examined pathologically and bacteriologically if necessary. In case of infectious diseases the result of the examination is returned promptly to the shipper with instructions and the necessary material for handling the outbreak. In the past year very few cases of glanders have been observed. This disease may be said to be extinct in Pennsylvania, and only occurs here and there as it is brought in from other States. Altogether there are about a dozen cases a year. A few cases of anthrax and some rabies have been reported. The rapid diagnosis of rabies as practised in the laboratory, consisting in an examination of the cervical ganglion, economizes a great deal of valuable time in cases of suspected rabies.

The instructions for obtaining specimens and the directions for handling contagious diseases sent to the veterinarians by the State Board and the Laboratory have been equivalent to a post-graduate course for all who have had to deal with such cases. It has stimulated the desire among our profession to read more and become more familiar with the most modern ideas. Its influence has done much to unify our profession and to make it more highly respected.

There are many factors at work for creating a greater demand for more and better domestic animals. This benefits our profession indirectly. Where valuable animals are kept skilful veterinarians are required. Among these factors the most notable are horse-shows, dog-shows, fat-stock shows, the numerous pet-stock shows, and county fairs. Each in its turn stimulates the desire to breed animals true to some certain type. The demand was never better for a first-class specimen of any class or type. In the last few years many wealthy gentlemen have become interested in show competitions, and fabulous prices are paid for animals that possess qualities suitable to become show animals. There has been a marked increase in the demand for saddle horses. The bicycle has been relegated to oblivion, and horseback riding has become much more popular. Horse-shows have increased the value of all fashionable classes of horses. These shows have been a source of education to those interested in the equine species.

In the matter of meat- and milk-inspection very little has been done in the past year. The Federal meat-inspectors are doing good work in Philadelphia, and we hope that the time is not far distant when we can have some system of meat-inspection for cities of the first and second class especially that will do as thorough work in the inspection of local dressed meat for home consumption as is practised at present by the Federal inspectors.

A very satisfactory system of milk-inspection is carried on in Philadelphia under the direction of the Pediatric Society. This society will recommend milk from any dairy that will conform to its requirements. The society employs a veterinarian, a bacteriologist, and a chemist, to make inspections at certain intervals at the expense of the dairymen. The veterinarian is to be satisfied with the health of the cows, with the cleanliness of the herd, stable assistants, milking utensils, general sanitary conditions, feed, etc., and that the interest and precautions necessary for producing clean milk are exercised faithfully and constantly. The tuberculin test is to be used at the discretion of the veterinarian.

The bacteriologist examines the milk at least once a month. The bacteria must not exceed 10,000 per c.c.

The chemist must be satisfied with the natural constituents of milk, and that no artificial preservatives are added to it.

At present there are five dairies producing milk for the Philadelphia market under these conditions. We hope in time that there will be milk enough produced under these or similar rigid conditions to supply all persons who appreciate the necessity of using clean milk. Milk produced in this manner must of necessity bring a few cents more per quart than milk produced and handled in a careless manner. People recognize different qualities of clothing, meats, vegetables, cigars, liquors, etc.; why should they not also recognize and appreciate the different qualities of milk and be willing to pay for the extra pains necessarily involved for obtaining it clean and wholesome? The beneficial effect of the dairies producing milk under the directions of the Pediatric Society is observed by other dairymen, and a decided improvement is noticed on the farms in close proximity to such dairies. The same interest produced in horses by horse-shows and in dogs by dog-shows can also be produced in dairies by model dairies. It should be our duty to encourage such undertakings in every way possible. It has been said of dairies producing milk under such careful conditions that they are short-lived; that the enthusiasm is soon lost, and that business drifts back into the same old rut. This has not been observed in respect to the work done for the Pediatric Society. Certified milk has been produced and sold in Philadelphia for the past five years, and the demand is gradually increasing.

The Veterinary Department of the University of Pennsylvania is at present located in temporary quarters. The old buildings were razed last year to make room for a medical and veterinary laboratory. The plans for the new building are about completed. The construction is expected to begin soon. It is to be located on one of the principal streets near the University, on a site that will be much more desirable than where it formerly stood. The plans are for one of the most complete and unique buildings of its kind in any English-speaking country.

Three members of our profession have died in the last year. Dr. W. T. Miller, of Apollo, Pa., one of the old members of the A. V. M. A., died June 18, 1902. He was a graduate of the American Veterinary College, class of 1887.

Dr. James Beatty, a native of Pennsylvania, graduated from the Veterinary Department of the University of Pennsylvania in 1897. He was employed three years prior to his death as a Federal meat-inspector, and was located in Philadelphia at the time of his death. His loss is keenly felt by his many friends, who esteemed and respected him for his loyalty, integrity, perseverance, and professional skill.

Dr. Rush Shippen Huidekoper, whose death I also have the sad duty to report, was one of the greatest builders of the veterinary medical profession in America. His death is a national loss, and is most keenly felt in his native State by those who knew him best. The honor of doing his memory justice at this meeting has fallen on one of his most faithful friends and fellow-workers. Suffice it to say that his sentiments are the sentiments of the entire profession.

C. J. MARSHALL.

### RESOLUTIONS.

### *Committee on Standard of Excellence and Soundness.*

*Resolved*, That a committee of three be appointed to formulate a standard of excellence and soundness for horses of various classes, and report at the next annual meeting of this Association.

### *Tuberculosis.*

WHEREAS, In the repression of tuberculosis of cattle it is of the highest importance that the disease shall, so far as possible, be confined to the localities already infested; and

WHEREAS, Every hindrance to the shipment of tubercular cattle into the United States, from one State to another, or from one farm or district to another, helps to confine the disease and to lessen the difficulties attending its final eradication; be it

*Resolved*, That this Association approves the establishment and maintenance of Federal and State laws and regulations to prevent the transfer of tubercular cattle from place to place, excepting in quarantine.

### *Clinics.*

WHEREAS, For several years it has been the custom to offer clinical demonstrations as a part of the programme of our annual meetings; and

WHEREAS, It has become apparent that these clinics are a source of great interest and profit to a large number of the members; therefore, be it

*Resolved*, That we favor the continuance and improvement of this feature of our meetings by including other than surgical cases and the exhibition of pathological specimens; and if it be necessary in order to enable the local committees of arrangement to better perfect these clinics and make them more worthy such a scientific body, it would be well for the Association to render additional assistance by appropriating such a sum of money as may be necessary to accomplish the purpose.

*Texas Fever.*

WHEREAS, The Cotton States Association of Commissioners of Agriculture, at their annual meeting, held in the city of Nashville, Tenn., August 26 to 28, 1902, passed a resolution providing for the appointment of a committee of three veterinarians connected with the Association to investigate the subject of Texas fever among cattle in the area now pronounced permanently infected with the disease, with the object of ascertaining what steps should be taken to render the territory free from infection, and also devise and recommend such measures as will result in the co-operation of the States affected, to the end that Texas fever may be permanently driven out of the infected district; and

WHEREAS, The above-named Association has named as members of such investigating committee three honored members of the American Veterinary Medical Association—Drs. Carey, of Alabama; Butler, of North Carolina, and Dalrymple, of Louisiana—gentlemen whose intimate acquaintance with the subject to be investigated is well known and respected by us; therefore, be it

*Resolved*, That we hereby congratulate the Cotton States Association of Commissioners of Agriculture upon the wisdom of their action and the country upon the prospects of an intelligent solution of the great problems connected with this disease, which has and is producing such hardship and pecuniary loss to our Southern States.

*State Associations.*

*Resolved*, That it is the sense of this Association that it is the duty of State Veterinary Medical Associations to make their conditions for membership conform with those of this Association.

*Intelligence and Education.*

*Resolved*, That the Committee on Intelligence and Education be instructed to investigate and report at the next meeting upon the organization and work of all American veterinary schools and State examining boards. Be it further

*Resolved*, That members of the faculties of the various veterinary schools and the members of the State examining boards shall be communicated with and given an opportunity to submit written or oral statements upon the subjects under investigation.

*On the Death of Members.*

WHEREAS, It has pleased Almighty God to remove from our midst Dr. Rush Shippen Huidekoper, of Philadelphia, on December 17, 1901, one of our most esteemed and beloved members, one who had given the greater part of his energetic life to the building and uplifting of the veterinary profession on this continent, and the advancement of the best interests of this Association, we feel that through his death the veterinary profession has suffered an irreparable loss and our Association one of its most active members; we not only deplore his death as a member of our Association, but we mourn the loss of a true and tried social friend; therefore, be it

*Resolved*, That this Association extends to his bereaved family the sincere sympathy of its members in their affliction; and be it further

*Resolved*, That these resolutions be entered in the records of this Association, and a copy sent to his family.

Whereas, It has pleased Almighty God to remove from our midst, during the past year, Dr. Robert J. Saunders, of West Roxbury, Massachusetts, one of the original members of this Association, a man self-sacrificing, conscientious, and studious, enjoying the good-will and respect of all who knew him; therefore, be it

*Resolved*, That this Association sincerely regrets his loss, and extends to his family its sympathy in their affiiction; and be it further

*Resolved*, That these resolutions be entered on the records of this Association and a copy be sent to his family.

Whereas, It has pleased Almighty God to remove from our midst during the past year Dr. Thomas F. Barron, of Baltimore, Maryland; therefore, be it

*Resolved*, That this Association regrets his loss and extends to his family its sympathy in their bereavement; and be it further

*Resolved*, That these resolutions be entered upon the records of this Association and a copy be sent to his family.

Whereas, It has pleased the Almighty to remove from our midst Dr. John Faust, a valued member of our Association, whose death occurred in the month of July, 1901; therefore, be it

*Resolved*, That this Association greatly regrets his loss, and extends to his family its sympathy in their bereavement; and be it further

*Resolved*, That these resolutions be entered upon the records of this Association and a copy be sent to his family.

Tait Butler, *Chairman.*

---

Tænigen. There is another vermifuge which is popular with canine practitioners. It is prepared from areca nuts, and is very active in killing and expelling the round worm (Lumbrici) in dogs, and when given in moderation is entirely safe. It seems to narcotise the worm, and requires the use of a brisk cathartic. It is strongly commended by Mr. C. S. Smith, M.R.C.V.S., Barnsley; Mr. H. Hall, M.R.C.V.S., Southampton, and several other canine practitioners. The dose of tænigen is from 15 to 60 minims, according to the size and body-weight of the dog. It is also best given in capsules.—*Ibid.*

